# De novo mutations of *TUBA3D* are associated with keratoconus

**DOI:** 10.1038/s41598-017-13162-0

**Published:** 2017-10-19

**Authors:** Xiao-dan Hao, Peng Chen, Yang-yang Zhang, Su-xia Li, Wei-yun Shi, Hua Gao

**Affiliations:** 1 State Key Laboratory Cultivation Base, Shandong Provincial Key Laboratory of Ophthalmology, Shandong Eye Institute, Shandong Academy of Medical Sciences, Qingdao, 266071 China; 2 Shandong Eye Hospital, Shandong Eye Institute, Shandong Academy of Medical Sciences, Jinan, 250021 China

## Abstract

Keratoconus (KC) is a common degenerative corneal disease, and heredity plays a key role in its development. Although few genes are known to cause KC, a large proportion of disease-causing genes remain to be revealed. Here, we report the identification of *TUBA3D* as a novel gene linked to KC. Using whole-exome sequencing of a twins pedigree, a novel de novo mutation (c.31 C > T, p.Gln11stop) in *TUBA3D* gene was identified. A screening performed in 200 additional unrelated patients with KC revealed another two mutations (c.201insTT, p.Val68Leufs*2; c.*2 G > A) in two patients. *TUBA3D* was expressed highly in the cornea, and the twins had lower *TUBA3D* expression and higher UPA and MMP1 expressions than the normal parents. Through function prediction and *in vitro* cell experiment, we further demonstrated that the mutant proteins of *TUBA3D* were unstable and could lead to human corneal fibroblast cells performing higher MMPs expression and oxidative stress. These changes thus reduce the amount of extracellular matrices within corneas and undoubtedly play a major role in stromal thinning, which is characteristic of KC corneas. Our study showed that *TUBA3D* is a new gene that causes KC, thus supporting the evidence that this protein has an additional function into the human cornea.

## Introduction

Keratoconus (KC) is a common degenerative corneal disease with a worldwide prevalence of approximately 1:2000^1,2^. Onset usually occurs during adolescence. KC can affect patients lifelong. KC is characterized by corneal ectasia, thinning, and a cone-shaped protrusion, thus resulting in reduced vision, irregular astigmatism, and corneal scarring^1^. It affects both genders and all ethnicities^2^. The limited availability of medical treatments makes KC become a significant clinical problem worldwide and a leading indication for corneal transplantation^3,4^.

Despite the visual and social effects of KC, its underlying biochemical and cellular basis is poorly understood. A large number of studies show a strong familial predisposition in KC development, and genetic factors play a key role in its development^5–7^. In most published studies, the inheritance pattern of KC is autosomal dominant with incomplete penetrance or variable expressivity^8–11^. Detection and identification of the pathogenic gene is both a research hotspot and a challenge in this field. To date, more than 20 genes/loci identified by genetic mapping or candidate gene screening have been reported to be associated with KC^5–7^. However, most of the results cannot be repeated in other studies. Only a small number of gene mutations were confirmed to be pathogenic, such as *VSX1*, *TGFBI*, and *mir184* ^7,12–14^. These reported pathogenic genes explain only a small percentage of KC^7,13,15^, and the etiology of most patients is still unknown. To gain further understanding of KC, we focused on patients who did not carry a defined genetic mutation in any of the previously reported genes.

Our group collected the pedigree of one pair of monozygotic twins. The twins had KC, but their parents did not. This situation suggested that the inheritance pattern of this family was either autosomal recessive or de novo mutation. Twins are an excellent material for genetic studies of human traits and diseases. This twin pedigree provides valuable samples and is an effective way to detect the pathogenic genes of KC. First, we excluded the reported candidate gene mutation by sequencing. Then, we conducted exome sequencing on four core members of this family (the twins and their parents) to find a new candidate gene. Through whole-exome sequencing (WES), we identified a de novo heterozygous nonsense mutation in a tubulin gene, tubulin alpha 3d *(TUBA3D)*. A screening of this gene in 200 patients with KC found additional two unrelated patients with different mutations in the *TUBA3D* gene. We further demonstrated that the mutant protein of *TUBA3D* was unstable and could lead to human corneal fibroblast cells (HTK) performing higher matrix metalloproteinases (MMPs) expression and oxidative stress. Our findings identify a new disease gene underlying KC and provide insight into *TUBA3D* dysfunction in human corneal degeneration.

## Results

### Clinical features

**Figure 1:**
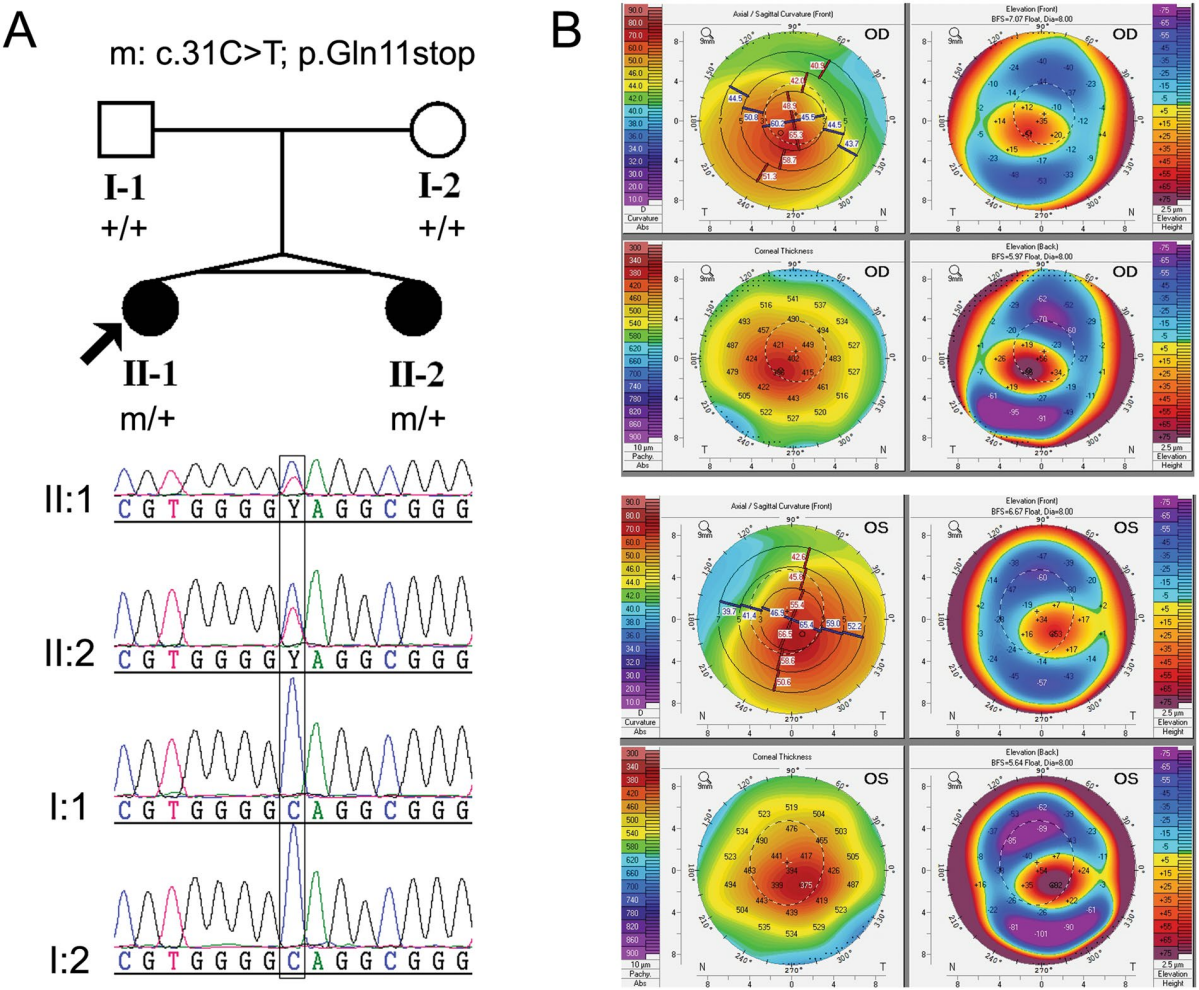
Pedigree and clinical characteristics of the twins’ family. (**A**) Pedigree of the twins’ family with *TUBA3D* mutation. The proband is indicated by an arrow. Sanger sequencing confirms the mutations identified by WES. (**B**) Videokeratograph of the proband. The results showed high central corneal curvature (i.e., Kmax and Kmin of the front surface >60D), significant protrusion of the front and posterior surfaces, and thinning of the corneal thickness of both eyes. OD, right eye. OS, left eye.

The proband and her twin sister, aged 23 years old, were born from an uneventful pregnancy. Seven years ago, the proband began vision loss with no cause. Five months ago, her visual acuity decreased significantly. She came to the hospital for medical treatment and was diagnosed with bilateral KC. Both her eyes had corneal ectasia, thinning, and a cone-shaped protrusion with Vogt’s striae and Fleischer’s ring. Signs of videokeratography showed the typical characteristics of KC (Fig. 1B). Clinical examination of her twin sister and parents showed that her twin sister was a KC patient too, but their parents were not. The pedigree of this family is shown in Fig. 1A, and it suggests that the inheritance pattern of the twins’ family was either autosomal recessive or de novo mutation.

### Exome sequencing detects a nonsense variant in *TUBA3D*

**Figure 2:**
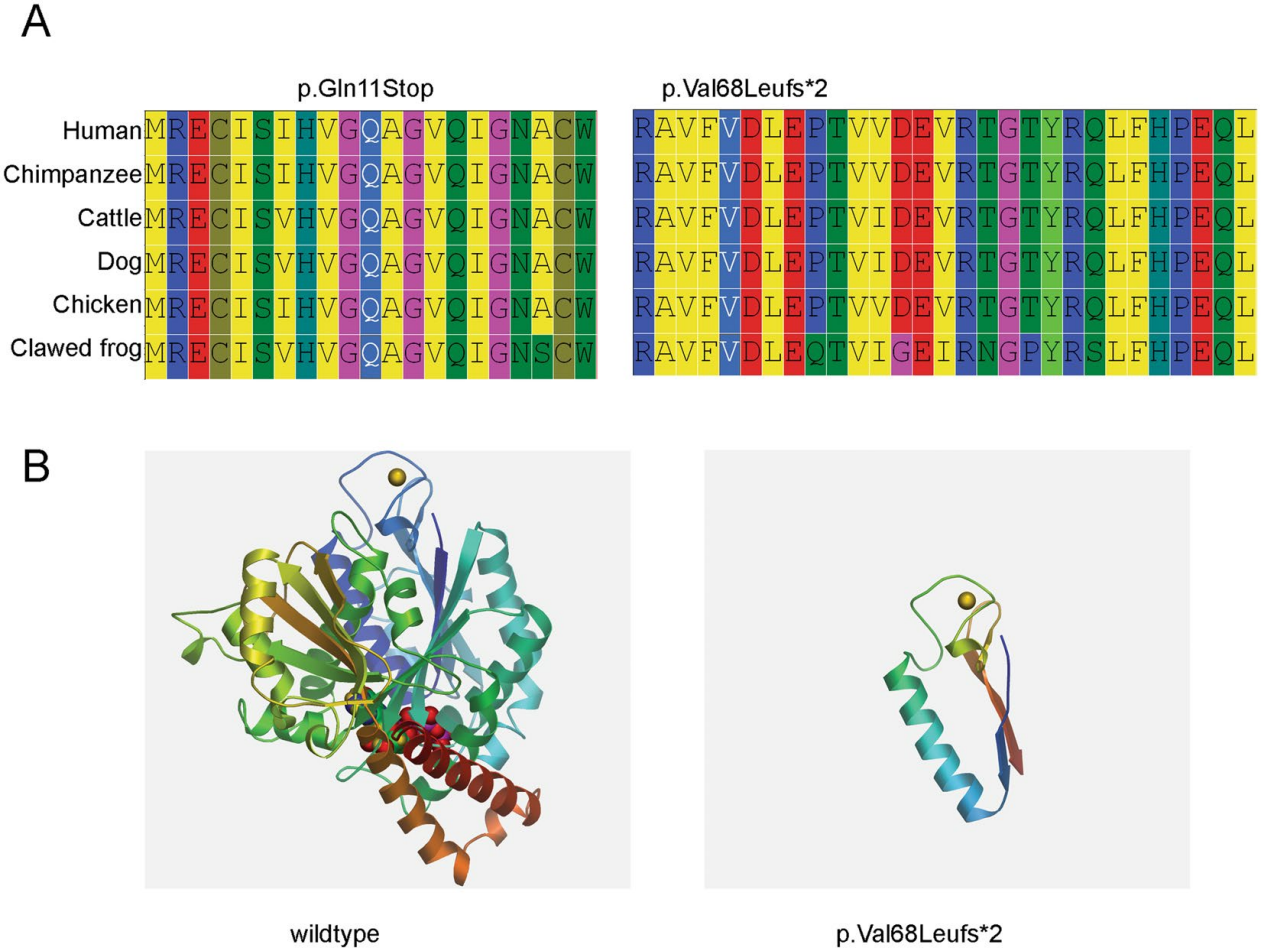
Conservative and tertiary structure prediction of mutant proteins. (**A**) Cross-species comparison of the region of *TUBA3D* indicates that the identified mutations affect highly conserved residues. Alignment of sequences surrounding the Gln11stop and Val68Leufs*2 mutations in Human (NP_525125.2), Chimpanzee (NP_001233579.1), Cattle (NP_001039875.1), Dog (XP_005640747.1), Chicken (XP_422851.1) and Clawed frog (NP_001081575.1). (**B**) The tertiary structure of the mutant and wildtype proteins predicted by the Swiss-Model workspace.

To reveal the underlying genetic defect in this family, we initially screened three reported candidate genes (*VSX1*, *TGFBI*, and *mir184*) using Sanger sequencing. No pathological mutation was found in any of these knows genes. Thus, we conducted WES on four core members of this family (the twins and their parents) to find a new candidate gene. The average sequencing depth and coverage of our analysis were 62.85× and 99.96%, respectively. With the data analysis and variants, as well as the filtering strategy described in the methods section, neither a gene with a homozygous variant nor a gene with a compound heterozygous variant was identified. To detect the de novo variations, we compared the sequencing results from the twins to their parents, and one gene (*TUBA3D*) with de novo variants was identified. Sanger sequencing was conducted to confirm the results obtained by WES. Finally, the de novo mutation (NM_080386.3: c.31 C > T, p.Gln11stop in *TUBA3D*) in the twins was confirmed by Sanger sequencing (Fig. 1A). This mutation was a true de novo mutation as it was absent in her parents (Fig. 1A). The mutation was not found in the 200 unrelated health controls and absent from the dbSNP (http://www.ncbi.nlm.nih.gov/projects/SNP/), HapMap, 1000 Genomes (http://www.1000genomes.org), NHLBI Exome sequencing project databases, and ExAC database. This variant was located in a highly conserved domain and led to a truncated protein (Fig. 2A). It was considered “disease causing” as predicted by Sorting Intolerant from Tolerant (SIFT)^16^ and Mutation Taster (Table 1)^17^. These findings suggest that it is a candidate mutation.

**Table 1:** Pathogenicity assessment of *TUBA3D* mutations.

Nucleotide change	Amino acid change	Predictions of pathogenicity		Frequency in patient alleles (Methods)	Frequency in control alleles (Methods)
		SIFT	Mutation taster		
c.31 C > T	p.Glu11Stop	Deleterious	Disease causing	Twins family (WES and Sanger sequencing)	0/200 (HRM)
c.201insTT	p.Val68Leufs*2	NA	Disease causing	1/200 (Sanger sequencing)	0/200 (HRM)
c.*2 G > A	3′UTR	NA	NA	1/200 (Sanger sequencing)	0/200 (Sanger sequencing)

NA, not application; HRM, high-resolution melting analysis.

### Mutational screening in additional KC patients

To examine the mutation frequency of *TUBA3D* in KC, we screened 200 unrelated sporadic KC patients. We identified two additional patients with KC who had heterozygous *TUBA3D* mutations (Table 1). One mutation was c.201insTT (p.Val68Leufs*2), which is an insertion mutation that changes the reading frame (i.e., grouping of the codons) and results in a completely different translation from the original. This mutation was predicted to alter highly conserved amino acids among different species (Fig. 2A) and considered “disease causing” as predicted by Mutation Taster (Table 1). It was also not found in 200 unrelated health controls and was absent from the dbSNP, HapMap, 1000 Genomes, and NHLBI Exome sequencing project databases. The other mutation was c.*2 G > A, which is located in the 3′UTR of *TUBA3D*, and it was absent in the 200 unrelated health controls.

To further identify the putative pathogenicity of these mutations, we computed the physico-chemical parameters and predicted the tertiary structure of mutant and wildtype proteins. The physico-chemical parameter changes of the mutant protein are shown in Table 2. Compared with the wildtype, the mutant proteins have a lesser number of amino acids, have a changed aliphatic index and hydropathicity, and are unstable (Table 2). The tertiary structures of mutant and wildtype proteins are illustrated in Fig. 2B. As the c.31 C > T mutant protein sequence is too short, its tertiary structures cannot be predicted. These additional mutations of *TUBA3D* in other patients and the predicted changing of mutant proteins support the finding that *TUBA3D* is a disease-causing gene.

**Table 2:** The physico-chemical parameters changes of mutant protein compared to wildtype.

Types	Number of amino acids	Molecular weight	Theoretical pI	Estimated half-life	Instability index	Aliphatic index	Grand average of hydropathicity
Wildtype	450	49959.5	4.98	30 hours (mammalian reticulocytes, *in vitro*)	stable	82.58	−0.185
c.31 C > T	10	1144.3	6.50	30 hours (mammalian reticulocytes, *in vitro*)	unstable	107.00	0.520
c.201insTT	92	9936.1	5.80	30 hours (mammalian reticulocytes, *in vitro*)	unstable	56.20	−0.300

Note: Theoretical pI is the pH at which a particular molecule carries no net electrical charge in the statistical mean; The half-life is a prediction of the time it takes for half of the amount of protein in a cell to disappear after its synthesis in the cell; Instability index provides an estimate of the stability of your protein in a test tube; The aliphatic index of a protein is defined as the relative volume occupied by aliphatic side chains (alanine, valine, isoleucine, and leucine); Grand Average of Hydropathy is calculated as the sum of hydropathy values of all the amino acids, divided by the number of residues in the sequence.

### *TUBA3D* expression analysis in human tissues

**Figure 3:**
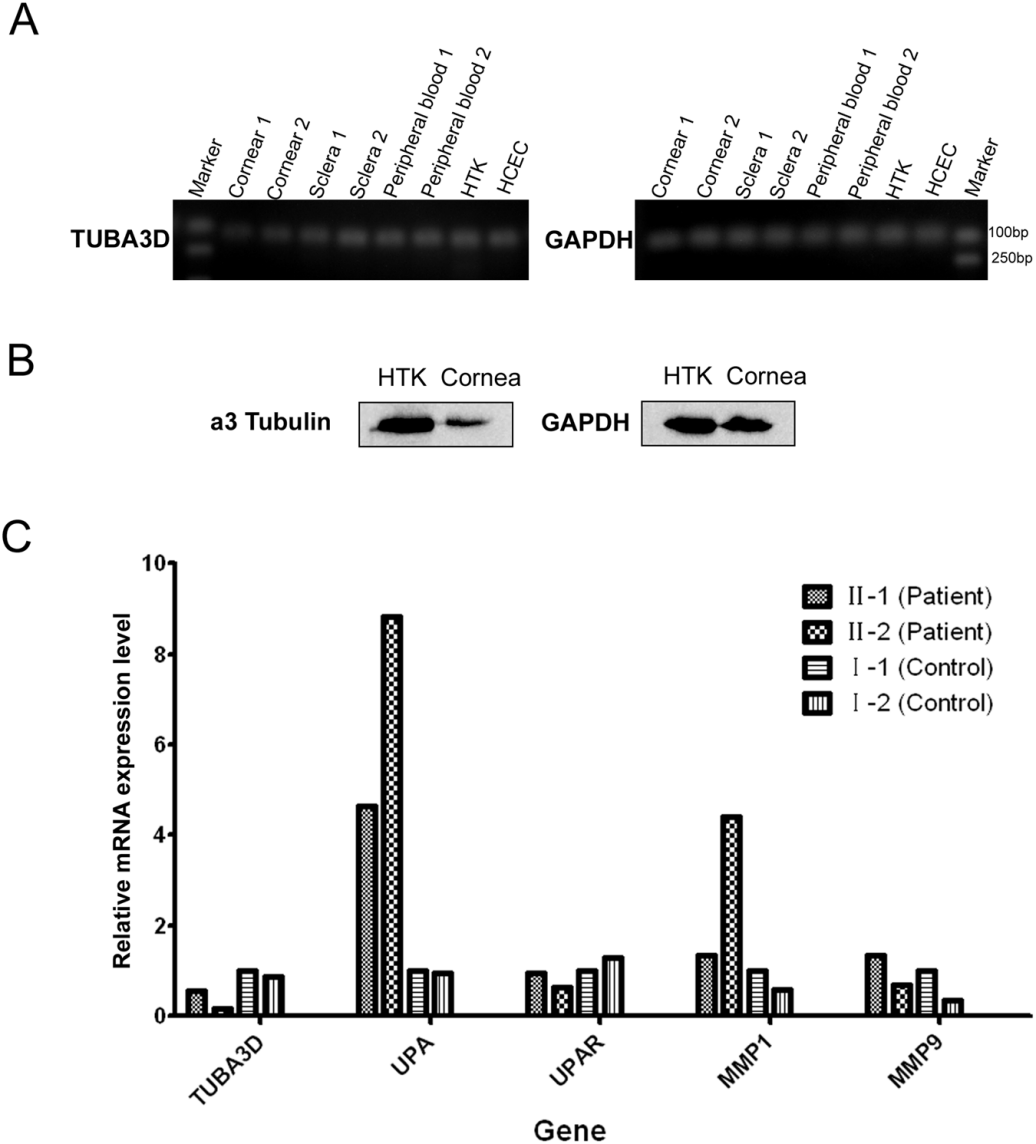
*TUBA3D* expression analysis in human tissues. (**A**) mRNA expression of *TUBA3D* in the human cornea, sclera, peripheral blood, human corneal fibroblast cells (HTK), and human corneal epithelial cells (HCEC). (**B**) a3 Tubulin and GAPDH expression analysis in HTK cell and cornea by Western blotting (cropped from different parts of the same gel). (**C**) mRNA expression of *TUBA3D* and genes *UPA*, *UPAR*, *MMP1*, and *MMP9* in the blood samples of the twins and their parents.

To determine whether or not *TUBA3D* was expressed in the cornea or other tissues and cell lines, we examined *TUBA3D* expression by PCR in human cornea, sclera, peripheral blood, human corneal epithelial cells (HCEC), and HTK. The agarose gel result showed that *TUBA3D* was expressed in all the above tissue and cell lines (Fig. 3A). Western blot analysis confirmed that *TUBA3D* was expressed in both cornea and HTK (Fig. 3B). We also examined the expressions of *TUBA3D* and MMP genes *UPA*, *UPAR*, *MMP1*, and *MMP9* in the blood samples of the twins and their parents. The results are shown in Fig. 3C. Compared with the normal parents, the twins had lower *TUBA3D* expression and higher *UPA* and *MMP1* expressions.

### The c.31 C > T (Gln11stop) and c.201insTT (Val68Leufs*2) mutations lead to *TUBA3D* degradation and higher MMPs expression

**Figure 4:**
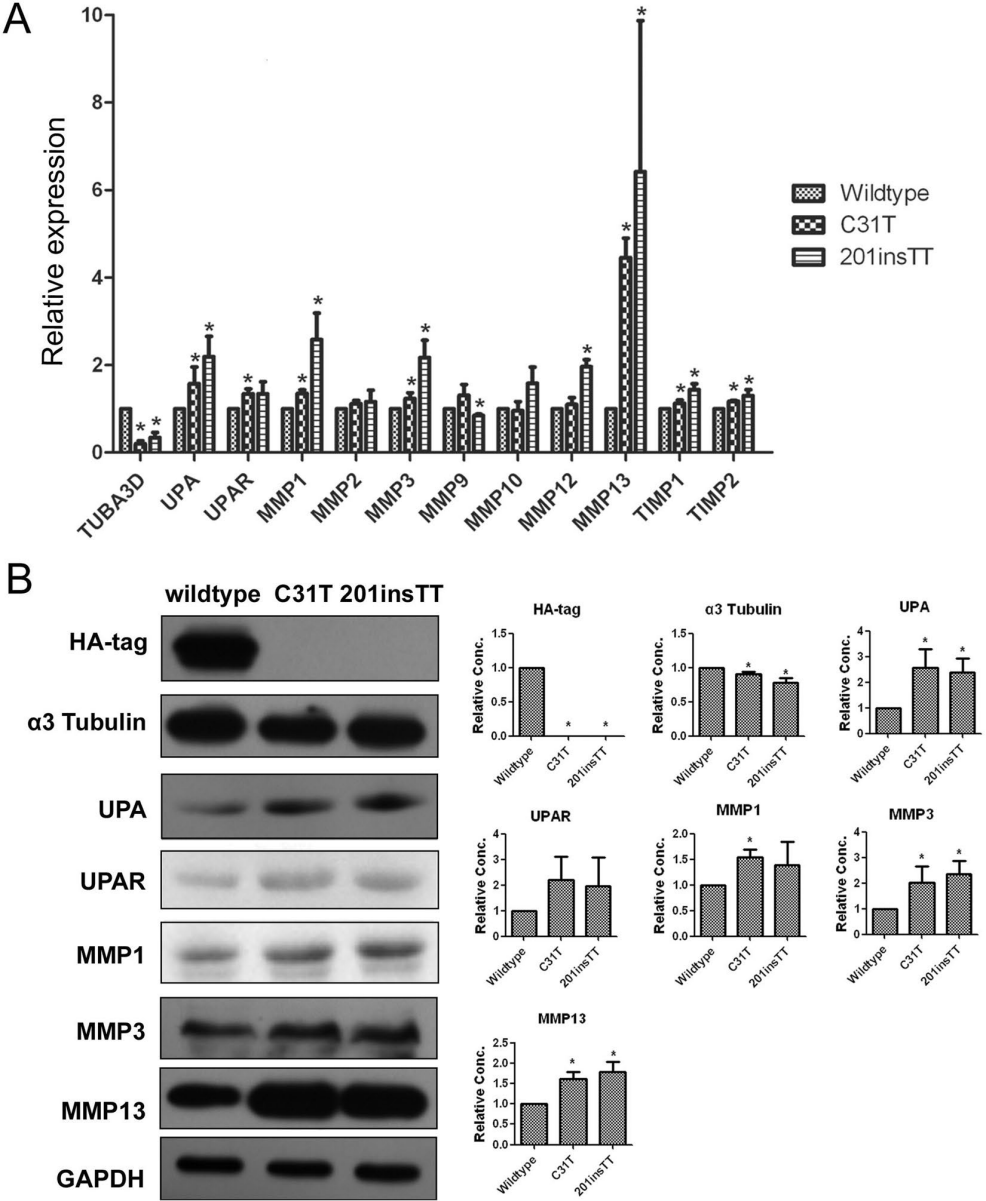
Expression analysis of *TUBA3D* and MMPs in wildtype, c.31 C > T (Gln11stop), and c.201insTT (Val68Leufs*2) mutation cells after 72 hours of transfection. HTK cells transiently transfected with constructs encoding HA-tagged wildtype and mutant (c.31 C > T, c.201insTT) *TUBA3D* proteins. (**A**) mRNA expression of *TUBA3D* and genes *UPA*, *UPAR*, *MMP1*, *MMP2*, *MMP3*, *MMP9*, *MMP10*, *MMP12*, *MMP13*, *TIMP1*, and *TIMP2* in HTK cells after transfection. n = 3. *P < 0.05. (**B**) HA, a3 Tubulin, UPA, UPAR, MMP1, MMP3, MMP13, and GAPDH expression analysis in HTK cells after transfection by Western blotting. Representative figures of Western blotting (left, cropped from different gels of the same samples) and the relative protein content of each group (n = 3) quantified by Image J software (right). *P < 0.05.

**Figure 5:**
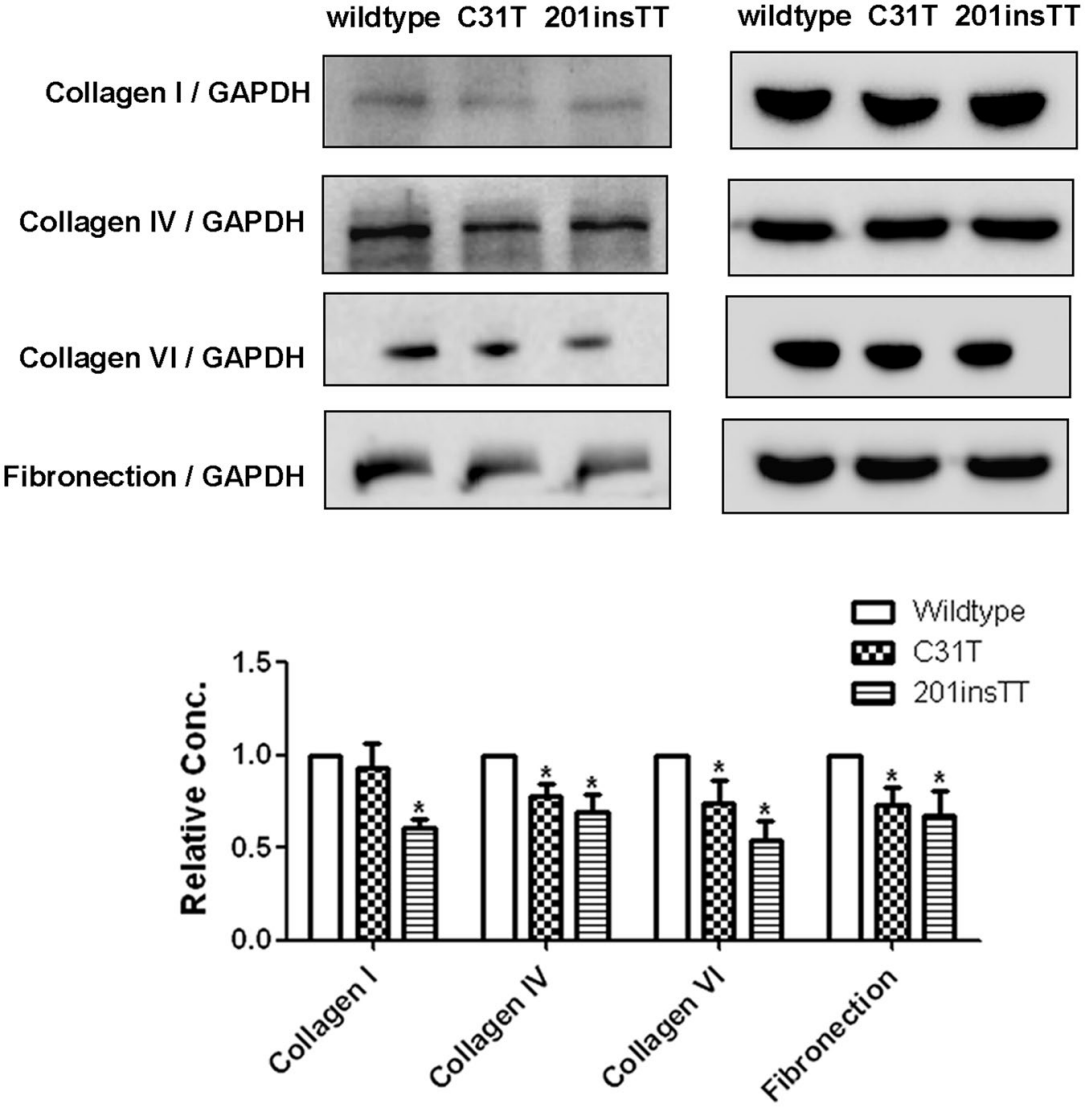
Expression analysis of ECM proteins in HTK cells after 72 hours of transfection by Western blotting. Representative figures of Western blotting (up, cropped from different gels of the same samples) and the relative protein content of each group (n = 3) quantified by Image J software (right). *P < 0.05.

**Figure 6:**
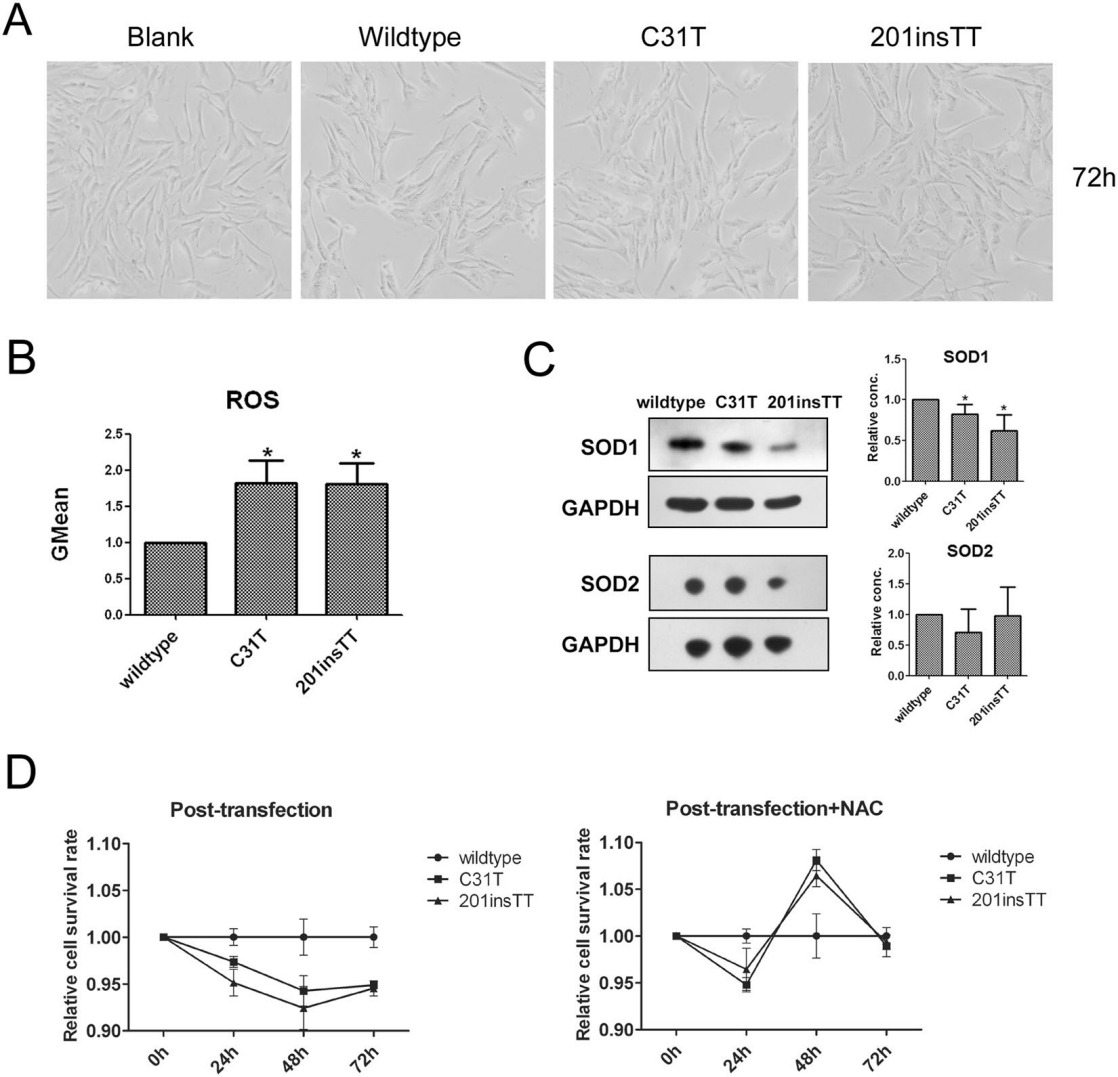
ROS and SOD1, SOD2 expression levels of the wildtype, c.31 C > T (Gln11stop), and c.201insTT (Val68Leufs*2) mutation cells. HTK cells transiently transfected with constructs encoding HA-tagged wildtype and mutant (c.31 C > T, c.201insTT) *TUBA3D* proteins. (**A**) The cell morphology after 72 hours of transfection (**B**) ROS levels in HTK cells after 48 hours of transfection. n = 3. *P < 0.05. (**C**) SOD1 and SOD2 expression analysis in HTK cells after transfection by Western blotting. Representative figures of Western blotting (left, cropped from different gels of the same samples) and the relative protein content of each group (n = 3) quantified by Image J software (right). *P < 0.05. (**D**) The cell survival analysis of the HTK cells after 24, 48, 72 hours of transfection with (right) and without NAC (left).

To examine whether or not the identified mutations affected *TUBA3D* stability, we constructed plasmids with c.31 C > T (Gln11stop) and c.201insTT (Val68Leufs*2) mutations expressed in HTK cells. Immunofluorescence (Supplementary Fig. S1) and Western blot (Fig. 4B) all revealed that wildtype-expressing cells detected the *TUBA3D*–HA fusion protein expression, but the c.31 C > T (Gln11stop)- and c.201insTT (Val68Leufs*2)-expressing cells showed no signal of HA, which suggested that these mutations affected *TUBA3D* stability and led to *TUBA3D* degradation. To test whether or not the mutant proteins affected the MMPs, we examined the expressions of genes *UPA*, *UPAR*, *MMP1 MMP2*, *MMP3*, *MMP9*, *MMP10*, *MMP12*, *MMP13*, *TIMP1*, and *TIMP2* using quantitative real-time PCR (qRT-PCR) (Fig. 4A) and Western blot (Fig. 4B). The results showed that c.31 C > T (Gln11stop)- and c.201insTT (Val68Leufs*2)-expressing cells had high *UPA*, *UPAR*, *MMP1*, *MMP3*, and *MMP13* expressions. Therefore, we further examined the extracellular matrix (ECM) protein (including Collagen I, IV, VI and Fibronectin) expression levels in HTK cells after transfecting with mutant *TUBA3D*. The results showed that Collagen IV, VI and Fibronectin protein levels drop slightly in c.31 C > T (Gln11stop)- and c.201insTT (Val68Leufs*2)-expressing cells compared with wildtype cells (Fig. 5). These results indicated that the mutant proteins of *TUBA3D* could lead to HTK cells performing higher MMP expressions and affect the extracellular matrix. However, there was no significant change in cell morphology after transfecting with mutant *TUBA3D* (Fig. 6A).

### The c.31 C > T (Gln11stop) and c.201insTT (Val68Leufs*2) mutations lead to HTK cells performing higher oxidative stress

To examine whether or not the identified mutations affect the oxidative stress of HTK cells, we measured the reactive oxygen species (ROS) and SOD1, SOD2 expression levels of the wildtype, c.31 C > T (Gln11stop), and c.201insTT (Val68Leufs*2) mutation cells. The results showed that c.31 C > T (Gln11stop) and c.201insTT (Val68Leufs*2) mutation cells had significantly higher ROS levels (Fig. 6B), lower SOD1 expression levels (Fig. 6C) than the wildtype cells. We also measured the cell survival of the HTK cells post transfection with and without antioxidant NAC (N-acetyl-L-cysteine). The relative cell survival rate of HTK cells were decreased after transfecting with mutant *TUBA3D*, and were recovered at 48 h, 72 h by adding NAC (Fig. 6D). These results suggested that the mutant proteins of *TUBA3D* could result in the decrease of SOD1 expression and lead to HTK cells performing higher oxidative stress, and affect cell survival.

## Discussion

In this study, using exome sequencing of a twin pedigree and directly screening 200 unrelated patients with KC, we identified *TUBA3D* as a novel gene linked to KC and found that it accounted for 1% of KC cases. To date, our functional studies provide initial evidence of the association of this gene with KC.

*TUBA3D* is located on chromosome 2q21.1, encodes a member of the alpha tubulin family, which is a major component of microtubules, and maintains cellular structure and function in intracellular transport^18^. This gene is highly expressed in normal human cornea, as indicated by qRT-PCR analysis, which suggests that *TUBA3D* could have an important role in the maintenance of corneal structure and function.

In this study, a de novo heterozygous mutation in the *TUBA3D* gene (c.31 C > T) was identified in the proband patient and her twin sister. By screening additional sporadic patients with KC, we identified two unique heterozygous mutations in *TUBA3D* (c.201insTT and c.*2 G > A) in two unrelated patients. None of these mutations was found in the 200 Chinese healthy controls. The mutations c.31 C > T and c.201insTT were novel based on their absence in all the databases. They were predicted to alter highly conserved amino acids among different species and considered “disease causing” as predicted by Mutation Taster. These mutations were also predicted to change the physico-chemical parameters and the tertiary structure of protein tremendously. These findings support *TUBA3D* as a disease-causing gene of KC.

Proteolytic phenomena contribute to the pathogenesis of KC, and MMPs were reported to be overexpressed in KC^19–21^. The identification of *TUBA3D* as a KC-causing gene raises the intriguing question of how mutations in a cytoskeleton gene that is expressed in the cornea could lead to a cornea-specific phenotype. *TUBA3D* encodes a cytoskeleton gene that has not been reported to be related to any other disease. To investigate the pathogenic mechanism of *TUBA3D*, we conducted function analysis through constructed plasmids with c.31 C > T (Gln11stop) and c.201insTT (Val68Leufs*2) mutations and expressed them in HTK cells. Our results showed that these mutations could affect *TUBA3D* stability and lead to *TUBA3D* degradation. Moreover, c.31 C > T (Gln11stop)- and c.201insTT (Val68Leufs*2)-expressing cells had higher MMPs *(UPA*, *UPAR*, *MMP1*, *MMP3*, *MMP13)* expressions and ROS levels than the wildtype cells. *TUBA3D* is a major component of microtubules, and it maintains cellular structure and function in intracellular transport^18^. Mutation of this gene could lead to protein degradation, which could affect the normal function of microtubules. Conversely, overexpressed MMPs could lead to proteolytic phenomena, which are a well-known pathogenesis of KC^19^. Oxidative stress was also reported to be involved in the pathogenesis of KC^22,23^. In summary, mutations of *TUBA3D* could lead to protein degradation, MMPs overexpression, and high oxidative stress, thus reducing the amount of extracellular matrices within corneas. These changes undoubtedly play a major role in stromal thinning and Bowman’s layer/basement membrane breaks, which are characteristic of KC corneas.

To the best of our knowledge, this study is the first to identify *TUBA3D* mutations in cases of KC. Our results indicate that the mutation frequency of *TUBA3D* in the population of Chinese patients with KC is 1.0% (two independent cases among 200). Our functional characterization also supports the hypothesis that *TUBA3D* is a new causative gene of KC and provides new insights into the molecular mechanisms underlying KC. Therefore, the mutational screening of *TUBA3D* should be considered for patients with KC to ensure proper molecular diagnosis.

## Methods

### Subject recruitment and clinical examination

The study was performed in accordance with the Declaration of Helsinki and approved by the Ethics Committee of Shandong Eye Institute (Qingdao, China). Written informed consent was obtained from all participants (or guardians). Patients diagnosed with KC were recruited from Qingdao Eye Hospital, Shandong Eye Institute (Qingdao, China). The diagnosis of KC was based on clinical examination (corneal stromal thinning, Vogt’s striae, Fleischer’s ring, Munson’s sign, signs of videokeratography, and refractive errors). In total, the family of one pair of monozygotic twins and 200 sporadic KC patients were collected. The pedigree (Fig. 1A) suggested that the inheritance pattern of these twins’ family was either autosomal recessive or de novo mutation. Two hundred unrelated healthy individuals of Chinese origin were used as control. Peripheral blood samples from all participating individuals were collected in EDTA tubes. Genomic DNA was extracted with Blood DNA Kit (Tiangen Biotech Co., Beijing, China).

### WES and data analysis

We conducted WES in all the members of this family (the twins and their parents) at Novogene (Beijing, China) to identify the causal gene. The SureSelect Human All ExonV5 Kit (Agilent Technologies, USA) was used for exome capture. The IlluminaHiseq. 2500 platform (Illumina Inc., San Diego, CA, USA) was employed for the genomic DNA sequencing of the twins and their parents.

The sequencing reads were mapped to the reference genome (UCSC hg19) using the Burrows–Wheeler Aligner software^24^. Samtools^25^ and Picard (http://broadinstitute.github.io/picard) were utilized to sort bam files and conduct duplicate marking to generate a final bam file, respectively. Samtools mpileup and bcftools were used to perform variant calling and to identify SNP/indels. ANNOVAR^26^ was used to conduct annotation. Variants were common (>0.5%) in dbSNP, HapMap, and the 1000 Genomes Project were filtered. Variants that were not present in any of the above databases were considered novel. Only SNPs and indels occurring in exons or located in canonical splicing sites were further analyzed, and nonsynonymous SNPs were submitted to SIFT^16^ and Polymorphism Phenotyping version 2 (PolyPhen-2)^27^, Mutation Taster^17^, and CADD^28^ for functional prediction. Among the four software programs, two showed that the variant was not benign and could be retained. Given the characteristics of the pedigree, homozygous, compound heterozygous, or de novo variations were considered to be candidate causal variations^29^.

### *TUBA3D* sequencing and genotyping

Sanger sequencing was performed to confirm and segregate the obtained results through whole-exome sequencing and to screen the *TUBA3D* gene in 200 unrelated sporadic KC patients. Primers were designed to amplify all five coding fragments and the intron–exon boundaries of the *TUBA3D* gene (NM_080386.3, see Supplementary Table S1). Every target fragment was amplified using Taq DNA polymerase (Takara, Dalian, China). The products were purified with alkaline phosphatase (Shrimp) (Takara, Dalian, China) and exonuclease I (Takara, Dalian, China), and subjected to direct DNA sequencing using the BigDye™ Terminator v3.1 Cycle Sequencing kit and ABI PRISM 3730 sequencer (Applied Biosystems Inc., USA). Sequences were aligned and analyzed using the DNASTAR software package (DNASTAR Inc., USA).

The novel mutations of *TUBA3D* were also genotyped in 200 unrelated healthy controls using high-resolution melt (HRM) analysis or Sanger sequencing. The primers used for genotyping the mutations of *TUBA3D* are shown in Supplementary Table S2. For HRM, the amplification assays were performed using the Type-it HRM PCR Kit (QIAGEN, Germany) on the Rotor-Gene Q Real-Time PCR system (QIAGEN, Germany). The results were obtained and analyzed using the Rotor-Gene Q Series Software (version 2.1.0). The Sanger sequencing method is the same as the above description.

### Protein structure and function prediction

Multiple protein sequence alignment among various species was carried out by MEGA software^30^. To further identify the putative pathogenicity of the variants, MutationTaster (http://www.mutationtaster.org/) was applied to test the possible effect of amino acid substitution on protein function^31^. The physico-chemical parameters of mutant and wild-type proteins were computed by ProtParam tool (http://web.expasy.org/protparam/). The tertiary structure of mutant proteins was predicted by the Swiss-Model workspace (http://swissmodel.expasy.org)^32^.

### Reverse transcription-PCR

Total RNA was prepared from venous blood (1 ml) of the family members using the RNA isolation kit for mammalian blood (Tiangen, Beijing, China). Total RNA was prepared from normal human corneas and cell samples using the NucleoSpin RNA II System (Macherey-Nagel, Duren, Germany). cDNA was synthesized from RNA using a commercial kit (PrimeScript^TM^ RT Reagent Kit (Perfect Real Time); Takara, Dalian, China). Expressions of the *TUBA3D* gene and the MMP genes (urokinase-type plasminogen activator, *UPA*; urokinase-type plasminogen activator receptor, *UPAR*; matrix metallopeptidase 1, *MMP1*; matrix metallopeptidase 2, *MMP2*; matrix metallopeptidase 3, *MMP3*; matrix metallopeptidase 9, *MMP9*; matrix metallopeptidase 10, *MMP10*; matrix metallopeptidase 12, *MMP12*; matrix metallopeptidase 13, *MMP13*; TIMP metallopeptidase inhibitor 1, *TIMP1*; and TIMP metallopeptidase inhibitor 2, *TIMP2*) were measured by qRT-PCR and normalized to glyceraldehyde-3-phosphate dehydrogenase (*GAPDH*). Primer sequences of the genes used for qRT-PCR are shown in Supplementary Table S3.

### *TUBA3D*–HA fusion constructs and expression in human corneal fibroblast cells

A telomerase-infected, extended-lifespan human corneal fibroblast cell line (HTK) were cultured in DMEM/F12 medium (Corning, USA) containing 10% fetal bovine serum (Gibco, USA) at 37 °C with 5% CO2. The wildtype, c.31 C > T (p.Glu11Stop), and c.201insTT (p.Val68Leufs*2) CDS sequences of the *TUBA3D* gene with the hemagglutinin (HA) tag at the C-terminal were synthesized directly and subcloned into the pcDNA3.1( + ) expression vector (Invitrogen, USA), respectively. The expression vectors were transfected in six-well plates using the Lipofectamine 2000 reagent (Invitrogen, USA) according to the manufacturer’s protocol. After transfection, the cells were collected at 72 or 48 hours to measure the mRNA/protein levels of HA, *TUBA3D*, MMPs and ECM proteins, or the ROS and related genes expression levels respectively.

### Immunofluorescence staining and flow cytometry analysis

For immunofluorescence staining, the cells were fixed in 4% paraformaldehyde in phosphate-buffered solution (PBS) for 15 min, followed by three PBS washes. The fixed cells were incubated with 0.1% Triton X-100 in PBS for 5 min, blocked in 5% BSA, and incubated with HA probe antibody (sc-805, Santa Cruz Biotechnology) overnight before incubation with the fluorescence-conjugated secondary antibody (Invitrogen, USA) for 1 hour. Images were obtained using an Eclipse TE2000-U confocal laser scanning microscope (Nikon, Tokyo, Japan) after counterstaining with 4′,6-diamidino-2-phenylindole. The ROS levels were measured using a Reactive Oxygen Species Assay Kit (Beyotime, China). Flow cytometry analysis was performed according to the manufacturer’s protocols, and the results were obtained. Cell survival of the HTK cells post transfection with and without antioxidant (NAC, 0.1 mM, Sigma-Aldrich) was measured using WST-1 Cell Proliferation and Cytotoxicity Assay Kit (Beyotime, China).

### Western blot

Total protein was prepared from each group using radioimmunoprecipitation assay (RIPA) buffer (Galen, Beijing, China) and quantified. Western blot analyses were performed as we described previously^33^. For each sample, the levels of proteins of interest were normalized to that of GAPDH. Primary antibodies included HA probe (sc-805, Santa Cruz Biotechnology), a3C Tubulin antibody (sc-134240, Santa Cruz Biotechnology), UPA antibody (ab169754, Abcam), UPAR antibody (ab103791, Abcam), MMP1 antibody (ab52631, Abcam), MMP3 antibody (ab52915, Abcam), MMP13 antibody (ab39012, Abcam), superoxide dismutase 1(SOD1) antibody (MABC684, Merck-Millipore), superoxide dismutase 2 (SOD2) antibody (ab68155, Abcam), collagen I antibody (ab138492, Abcam), collagen IV antibody (ab6598, Abcam), collagen VI antibody (ab182744, Abcam), fibronectin antibody (ab32419, Abcam) and anti-GAPDH antibody (KC-5G5, Kangchen, Shanghai, China). Raw data of representative blots were shown in Supplementary Figure S2.

## Electronic supplementary material

Supplementary Materials
