# Supplementary material for: De novo mutations of TUBA3D are associated with keratoconus
Source: Sci Rep. 2017 Oct 19;7:13570. doi: 10.1038/s41598-017-13162-0 (PMC5648796; doi:10.1038/s41598-017-13162-0)

# De novo mutations of *TUBA3D* are associated with keratoconus

Xiao-dan Hao, Peng Chen, Yang-yang Zhang, Su-xia Li, Wei-yun Shi, Hua Gao

**Figure S1. Representative figures of immunofluorescence staining of the *TUBA3D*–HA fusion protein in the wildtype, c.31C>T (Gln11stop), and c.201insTT (Val68Leufs*2) mutation cells after 72 hours of transfection.** HTK cells transiently transfected with constructs encoding HA-tagged wildtype and mutant (c.31C>T, c.201insTT) *TUBA3D* proteins. The anti-HA antibody stains the *TUBA3D* fusion proteins (left), and DAPI stains the cell nuclei (middle). Merged pictures are shown on the right. Magnification: 400×.

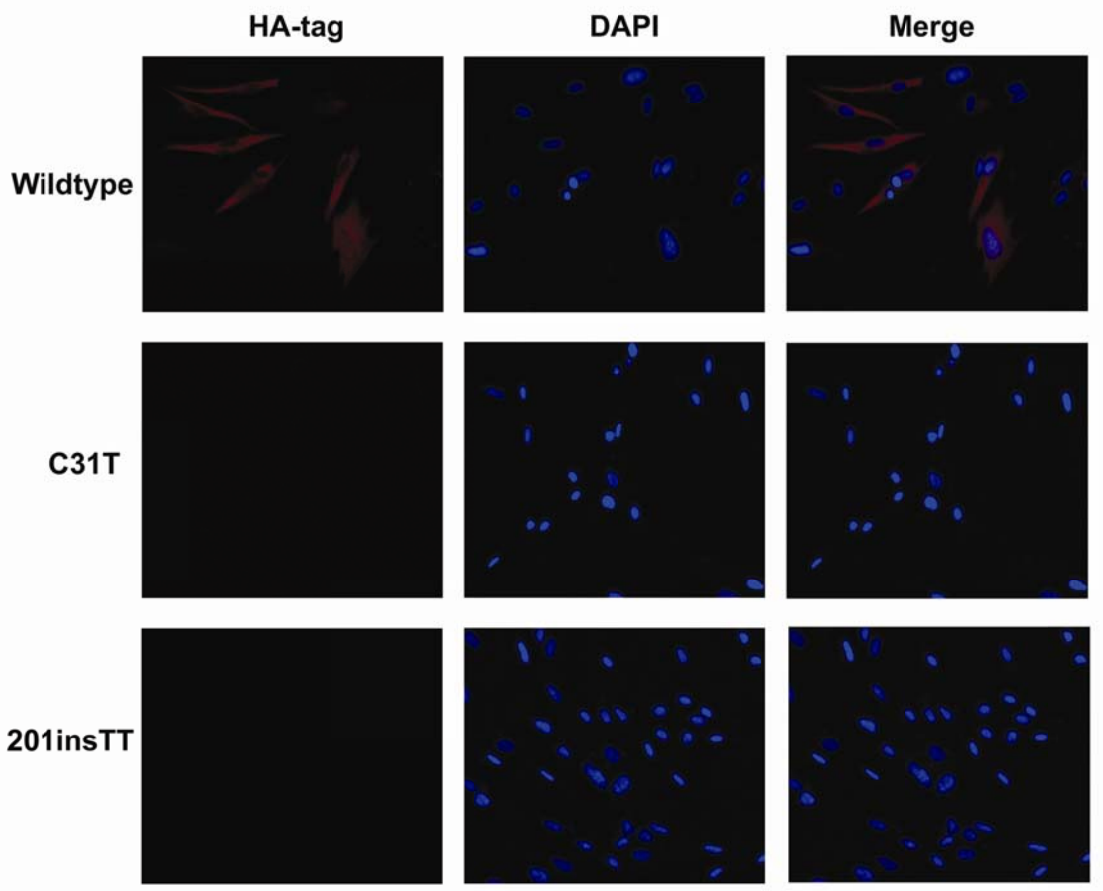

**Table S1 Primers used for amplifying five coding fragments of *TUBA3D***

| Amplified fragment | Forward | Reverse |
|---|---|---|
| Exon 1 | 5’-GAGCGTCCCCAGTACACA-3’ | 5’-TGTTTCGAGTAGCACAAAGAC-3’ |
| Exon 2 | 5’-TTTCCTTACCCAACCCGACT-3’ | 5’-AGGCACGCACGAGGGA-3’ |
| Exon 3 | 5’-TCCCTCGTGCGTGCC-3’ | 5’-TCACATTCCAAGAACTACCCCT-3’ |
| Exon 4 | 5’-GCATCTTGAGTCCTACTGAG-3’ | 5’-GTGATGACTCAGACAGAGAG-3’ |
| Exon 5 | 5’-CTCAGGTCATGTTATGCCCG-3’ | 5’-GAATGCTGTGCAGAACTCGGTTA-3’ |

**Table S2 Primers used for genotyping the mutations of *TUBA3D***

| Mutations | Forward | Reverse |
|---|---|---|
| C.31C>T | 5’-GGGTTCACTTTTATGTTCCTGTTC-3’ | 5’-TACAGTTCCCAGCAGGCATT-3’ |
| C.201insTT | 5’-GGGGACGACTCCTTCAACA-3’ | 5’-CCTCTCAGGAAAGCTGCCAT-3’ |
| C.*2G>A | 5’-CTCAGGTCATGTTATGCCCG-3’ | 5’-GAATGCTGTGCAGAACTCGGTTA-3’ |

**Table S3 Primer sequences of the genes used for RT-PCR**

| Genes | Forward | Reverse |
|---|---|---|
| *TUBA3D* | 5’-GAGCCCTACAACTCCATCCTG-3’ | 5’-TGGTGTACGTGGGACGTTCA-3’ |
| *UPA* | 5’-TACTGCAGGAACCCAGACAA-3’ | 5’-AGTCATGCACCATGCACTCT-3’ |
| *UPAR* | 5’-GGATCCAGGAAGGTGAAGAA-3’ | 5’-AGGTGTCGTTGTTGTGGAAA-3’ |
| *MMP1* | 5’-ACATGAGTCTTTGCCGGAGG-3’ | 5’-ATCCCTTGCCTATCCAGGGT-3’ |
| *MMP2* | 5’-TGGATGATGCCTTTGCTCGT-3’ | 5’-TATCCATCGCCATGCTCCCA-3’ |
| *MMP3* | 5’-AGGCAAGACAGCAAGGCATA-3’ | 5’-AGGTTCATGCTGGTGTCCTC-3’ |
| *MMP9* | 5’-CTTTGAGTCCGGTGGACGAT-3’ | 5’-TCGCCAGTACTTCCCATCCT-3’ |
| *MMP10* | 5’-GTGGAGTTCCTGACGTTGGT-3’ | 5’-AGTGGAGTCACCTCTTCCCA-3’ |
| *MMP12* | 5’-CCCGTATGGAGGAAACATTATAT-3’ | 5’-CCGGATTGCGTAGTCAACA-3’ |
| *MMP13* | 5’-TGACCCTTCCTTATCCCTTG-3’ | 5’-ATACGGTTGGGAAGTTCTGG-3’ |
| *TIMP1* | 5’-TGCACAGTGTTTCCCTGTTT-3’ | 5’-AGGATTCAGGCTATCTGGGA-3’ |
| *TIMP2* | 5’-AGAGAAGTGACGGCTCCTGT-3’ | 5’-CTGTTTCCAGGAAGGGATGT-3’ |
| *GAPDH* | 5’-ATGCTGGCGCTGAGTACGT-3’ | 5’-AGCCCCAGCCTTCTCCAT-3’ |

Figure S2. Raw data of representative gels and blots. Western blots were imaged with film in a darkroom or acquired using the ChemiDoc Touch Imaging System (Bio-Rad).

A, Original image of figure 3A.

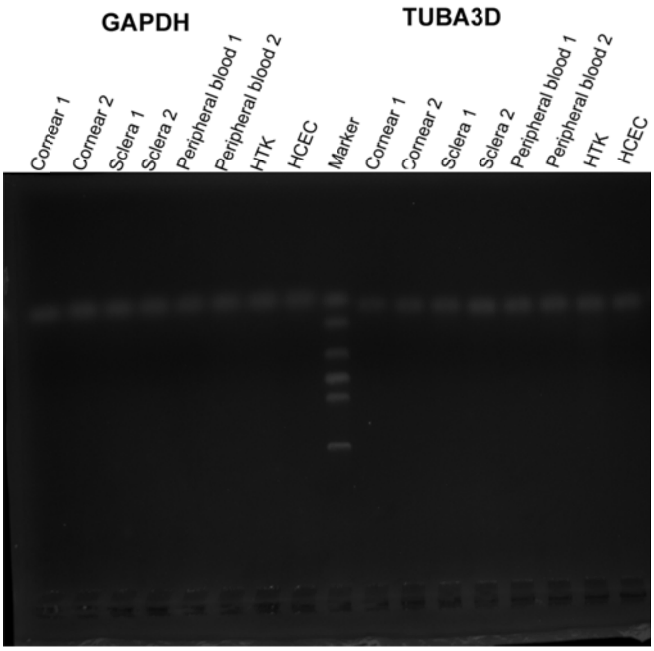


B, Original image of figure 3B.

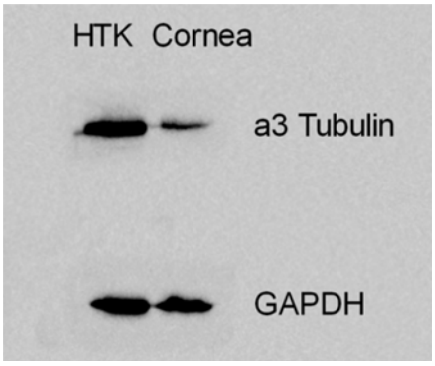


C, Original images of figure 4B. Lane1: wildtype; Lane 2: C31T; Line 3: 201insTT.

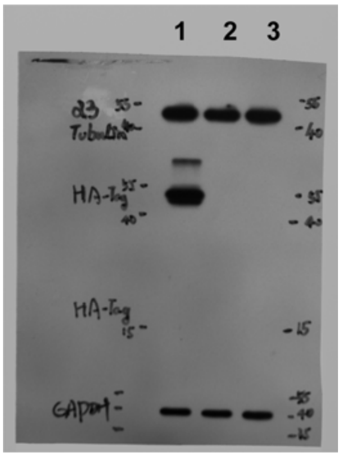


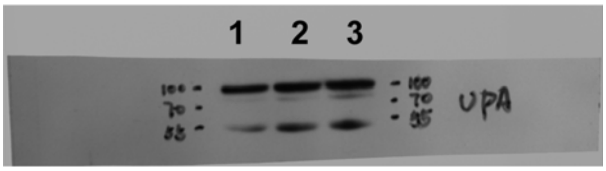

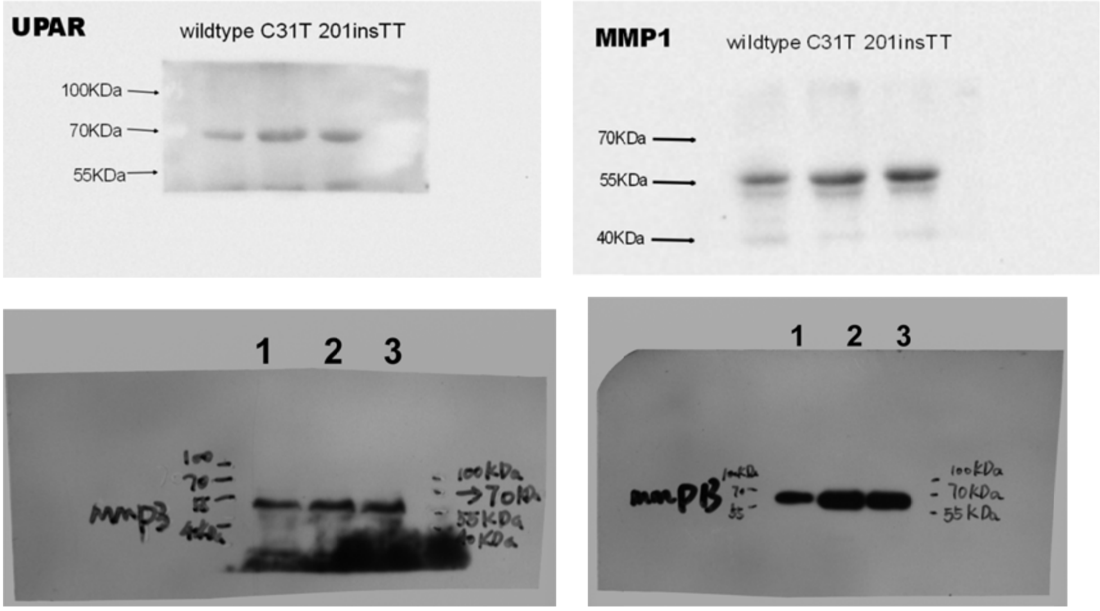


D, Original images of figure 5. Lane1: wildtype; Lane 2: C31T; Line 3: 201insTT.

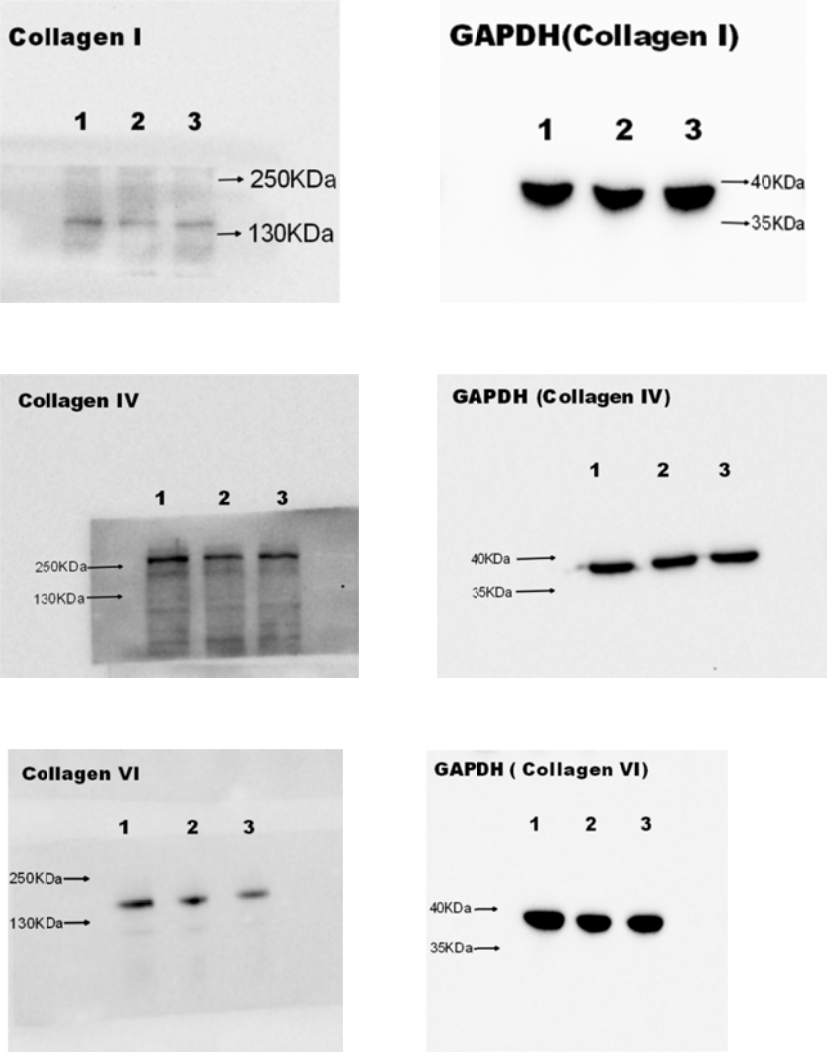

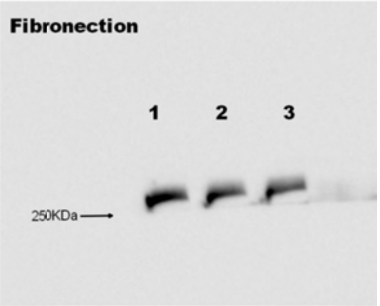


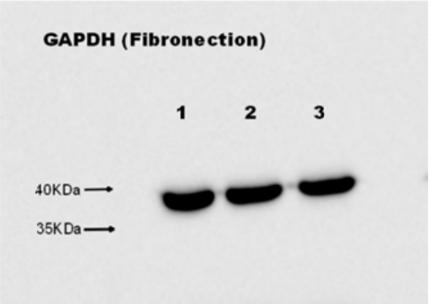


E, Original images of figure 6C.

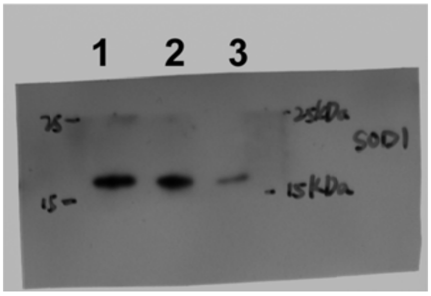


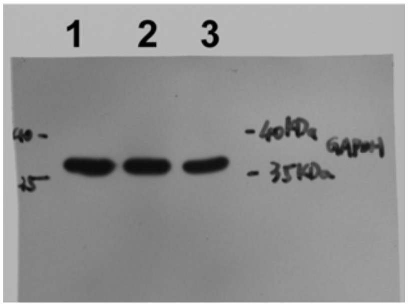


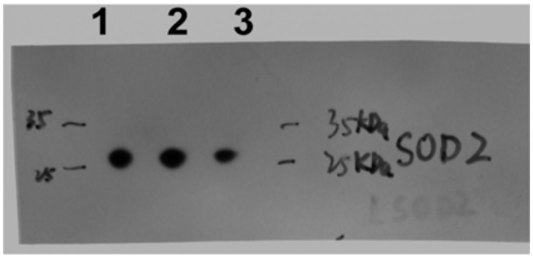


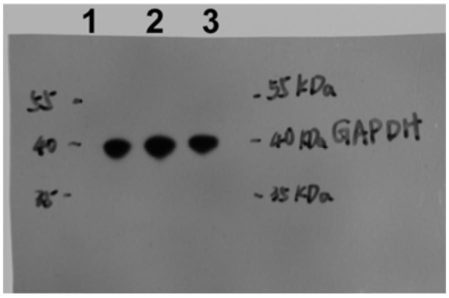

Supplement: Supplementary file 1 — Supplementary Materials [file 41598_2017_13162_MOESM1_ESM.pdf]
